# Zinc Fortification and Supplementation to Reduce Diarrhea in Children: A Literature Review

**DOI:** 10.3390/diseases13110380

**Published:** 2025-11-20

**Authors:** Sehar Iqbal, Zoha Imtiaz Malik, Maher Al Dabbas, Ishmal Akhtar, Aya Hussein

**Affiliations:** 1 College of Pharmacy, Al-Ain University, Abu Dhabi Campus, Abu Dhabi P.O. Box 112612, United Arab Emirates; ishmal5655@gmail.com (I.A.); ayahuusein@gmail.com (A.H.); 2 AAU Health and Biomedical Research Center, Al Ain University, Abu Dhabi P.O. Box 11111, United Arab Emirates; 3 Department of Human Nutrition and Dietetics, NUST School of Health Sciences, National University of Sciences & Technology (NUST), Sector H-12, Islamabad 44000, Pakistan; zoha.imtiaz@nshs.nust.edu.pk; 4 Department of Nutrition and Food Technology, Faculty of Agriculture, University of Jordan, Amman 11942, Jordan; m.aldabbas@ju.edu.jo

**Keywords:** zinc, fortification, supplementation, diarrhea, children

## Abstract

Background: Zinc deficiency is a major global health issue and appears to be responsible for risk of diarrhea and death, particularly in children under 5 years. This review therefore aimed to summarize the existing literature related to zinc supplementation and fortification for the prevention of diarrhea. Methods: In this literature review, we discussed the zinc-related biochemistry and pathophysiology of diarrhea and role of zinc in reducing the risk of diarrhea in children. Moreover, this literature review particularly analyzed studies published between 2014 and 2025, including systematic reviews, meta-analyses, and randomized controlled trials focusing on zinc fortification and supplementation for the prevention of childhood diarrhea. The studies covered a range of zinc dosing regimens (5–20 mg daily), preventive and therapeutic approaches, and combined interventions in children. Main outcomes such as diarrhea duration, severity, recurrence, growth, and side effects were assessed across diverse low- and middle-income populations. Results: Routine zinc supplementation helps to reduce all-cause diarrhea and respiratory infections. Zinc supplementation consistently reduced both the duration and severity of diarrhea in children. Also, diarrhea episodes and frequency were reduced in children taking zinc supplementation. Lower doses (5–10 mg) were mostly recommended to reduce vomiting. Combined zinc and vitamin A supplementation further improved outcomes, while long-term low-dose zinc supplementation prevented diarrhea and infections. Conclusion: This review confirms that zinc supplementation and fortification are effective, affordable strategies for reducing childhood diarrhea. Supplementation during diarrheal episodes reliably shortens duration and severity, with long-term benefits lasting for months. Continued research and integrated approaches for dosing and delivery are needed for both low- and middle-income countries.

## 1. Introduction

Zinc deficiency represents a significant and ongoing global health challenge, particularly for populations living in low- and middle-income countries [1]. It is estimated that 17% of the global population is at risk of inadequate zinc intake, with the highest prevalence rates noted in Africa (24%), and Asia (19%) [2]. The prevalence of zinc deficiency differs by age, and children under five from low- and middle-income countries (LMICs) have the highest rates of zinc deficiency, which is further associated with malnourishment and childhood comorbidities. As of 2020, 22% children under five reportedly had zinc deficiency, which, despite being much less than the 40% global prevalence in 1990, is still alarming enough to warrant serious action for better perinatal health [3]. Nearly 400 million children under five are impacted by zinc deficiency outcomes, highlighting the scale and urgency of the problem. Globally, more than 1 billion people are classified as zinc-deficient, and this deficiency is associated with serious consequences for child health, including growth retardation and impaired intellectual development [4].

The consequences of zinc deficiency are particularly severe when considering its relationship with childhood diarrhea, which remains one of the leading causes of illness and death among children worldwide. While the global burden of diarrhea among children under five has declined significantly from 1990 to 2021, mortality rates remain unacceptably high, especially in resource-limited settings where access to adequate nutrition and healthcare is limited [4]. Zinc deficiency is a major contributor to this burden, being responsible for approximately 116,000 child deaths each year due to its role in increasing susceptibility to diarrheal diseases [5]. The impact of diarrhea is not uniform across the globe; regions with lower socio-demographic indices experience much higher rates of both incidence and mortality. This disparity underscores the urgent need for targeted interventions and resources in the most affected areas to combat both zinc deficiency and its consequences [6].

Recent evidence has highlighted that the impacts of zinc deficiency are exacerbated in the presence of increased intestinal epithelial turnover. Pathological conditions such as gut infections and inflammation, and intestinal injuries increase zinc demand, as it is required for epithelial cell proliferation, mucosal barrier repair and restoration, and ensuring tight junction integrity [7]. Simultaneously, inflammatory processes reduce zinc absorption via increased intestinal permeability, zinc transporter dysregulation, and triggering of endogenous zinc losses, which compromise bodily zinc status [8]. This results in a bidirectional pathway in which diarrhea and enteral infections increase zinc absorption, while simultaneously decreasing its absorption, which then leads to recurrent diarrheal episodes [9]. Additionally, low zinc levels are further associated with taste distortion, including hypogeusia and dysgeusia. Zinc is an essential nutrient for taste regulation as it is needed for several enzymes and gut signaling pathways, and its deficiency can disrupt these mechanisms. This then results in reduced appetite, leading to decreased zinc intake, thereby creating an additional pathway for zinc deficiency [10].

Despite significant progress in reducing childhood diarrhea mortality, zinc deficiency remains a persistent threat to child health. It continues to compromise immune function and increase vulnerability to diarrheal diseases, particularly in malnourished populations where the effects are most pronounced [11]. Zinc deficiency is implicated in 14.4% of diarrhea deaths, 10.4% of malaria deaths, and 6.7% of pneumonia deaths among children aged six months to five years [4]. The ongoing high prevalence of zinc deficiency in these vulnerable groups, combined with the continued burden of childhood diarrhea, highlights the need for comprehensive and sustained intervention strategies that go beyond short-term solutions and address the root causes of micronutrient deficiencies [12].

Current approaches to combat zinc deficiency and its consequences include therapeutic zinc supplementation during diarrheal episodes, preventive supplementation programs, and food fortification initiatives [5]. The World Health Organization and UNICEF recommend that children with acute diarrhea receive 20 mg of zinc daily for 10–14 days, and infants under six months receive 10 mg per day [11]. Numerous studies have confirmed the effectiveness of zinc supplementation in reducing both the duration and severity of diarrhea in children, supporting its inclusion in standard treatment protocols and public health guidelines [12]. Moreover, a variety of international strategies have been implemented, including large-scale food fortification programs and zinc supplementation initiatives to address these challenges. As of August 2022, 82 low- and middle-income countries had established mandatory standards for fortifying at least one staple food with essential nutrients, including zinc. The introduction of high-quality, mandatory fortification programs that include zinc has the potential to reduce the global prevalence of inadequate zinc intake by up to 50% [5]. These efforts demonstrate a growing recognition of the importance of addressing micronutrient deficiencies as part of broader public health strategies [13].

Despite the strong evidence base for zinc supplementation, important gaps are still persistent and need a comprehensive approach. There are still uncertain findings about the optimal dosing strategies, the most effective delivery mechanisms, and the best approaches for long-term prevention, especially in diverse and resource-limited settings [14]. Recent research suggests that lower doses of zinc may be just as effective as higher doses, with the added benefit of fewer side effects, such as vomiting, which can improve adherence and outcomes [12], but conclusive evidence is still missing. Furthermore, there is a need for more research comparing the effectiveness of different intervention strategies, including the relative benefits of food fortification versus direct supplementation, to ensure that programs are both effective and sustainable in the long term [15]. Given these ongoing challenges and the evolving landscape of public health interventions, the aim of this literature review is to synthesize current and comprehensive evidence on the role of zinc in the human physiology and pathophysiology of diarrhea, particularly in children. The review also seeks to evaluate the effectiveness of various intervention strategies about zinc fortification and supplementation to enhance immunity and to reduce diarrhea in children. Moreover, we have highlighted the related gaps in knowledge and practice to identify optimal approaches for zinc fortification in diverse global settings to achieve better health outcomes for children. For this purpose, we have summarized the literature discussing zinc biochemistry, the nutritional importance of zinc, the pathophysiology of diarrhea, the role of zinc in reducing the risk of diarrhea, zinc dosage, and administration strategies for diarrhea treatment in children. Moreover, we have included all the studies published between 2014 and 2025 focusing on zinc fortification and supplementation for the prevention of childhood diarrhea.

## 2. Zinc: Biochemistry and Nutritional Role

### 2.1. Biological Role of Zinc in Child Growth and Immunity

Zinc is an essential trace mineral that plays a central and multifaceted role in human physiology. The role is particularly crucial in children, as shown in Table 1, where it is required for proper growth, immune defense, and cellular function [11]. At the molecular level, zinc is involved in a wide array of biological processes, acting as a structural, catalytic, and regulatory ion in hundreds of enzymes and proteins. It is fundamental in the regulation of inflammatory pathways, helping to maintain oxidative balance and modulate immune function. Zinc’s anti-inflammatory properties are largely mediated by its ability to inhibit the activation of Nuclear Factor-kappa B (NF-κB), a key transcription factor that drives the production of pro-inflammatory cytokines. By modulating NF-κB activity and reducing histamine release from basophils, leukocytes, and mast cells, zinc helps to control inflammation and prevent excessive immune responses that can damage tissues [5].

Beyond its anti-inflammatory effects, zinc is crucial for antioxidant defense. It is a cofactor for enzymes such as superoxide dismutase, which neutralizes reactive oxygen species (ROS) and protects cells from oxidative stress. Zinc deficiency has been shown to increase oxidative damage and the generation of inflammatory cytokines, while supplementation can upregulate protective factors like A20 and IKBα, further inhibiting inflammatory signaling [16,17]. This antioxidant role is especially important in rapidly growing children, whose tissues are particularly susceptible to oxidative injury.

Zinc is also indispensable for protein and lipid processing, cell integrity, and the maturation of various cell types. It is involved in the metabolism of macronutrients and the synthesis and breakdown of nucleic acids, proteins, and lipids [11]. In pediatric populations, zinc’s role in growth and development is especially pronounced and deficiency can lead to stunted growth, impaired cognitive development, and increased susceptibility to infections [18].

The immune system is highly dependent on adequate zinc status for both innate and adaptive responses. Zinc ions are involved in the regulation of intracellular signaling pathways in immune cells, affecting the formation, activation, and maturation of lymphocytes, as well as the function of neutrophils and natural killer cells [16]. Zinc homeostasis is tightly controlled by specialized transporters (ZIP and ZnT families) and binding proteins, ensuring that immune cells have sufficient zinc to mount effective responses [19]. Disruptions in zinc homeostasis can impair cytokine production, antibody formation, and the ability of immune cells to clear pathogens, leading to increased vulnerability to infections and inflammatory diseases.

Another important function of zinc is its involvement in the development and functioning of thymulin, a thymic hormone essential for T-cell maturation [20]. Zinc deficiency leads to decreased thymulin activity, resulting in impaired differentiation and function of T lymphocytes, which are critical for adaptive immunity. This has been demonstrated in both human and animal studies, where zinc supplementation restores thymulin activity and corrects immune defects [19]. Furthermore, zinc contributes to the regulation of apoptosis, or programmed cell death, which is necessary for the removal of damaged or infected cells and the maintenance of immune homeostasis. Zinc can suppress the activation of caspases, a family of enzymes that drive apoptosis, thereby protecting healthy cells during immune responses [18].

### 2.2. Zinc Mechanism of Action

Zinc plays a therapeutic role in childhood diarrheal diseases through multiple interconnected pathways, comprising molecular, immunological, and gut microbiota mechanisms, as shown in Table 2. It acts as an essential cofactor for various enzymes and is a structural component of zinc-finger transcription factors that are responsible for vital cellular functions such as cell proliferation, apoptosis, and DNA synthesis. Therefore, zinc plays essential roles in mucosal regeneration after cellular injury. These roles make zinc a vital component of epithelial regulation and integrity maintenance. Zinc acts to stabilize the enterocytes’ membranes, which reduces intestinal permeability, and simultaneously promotes restoration and proliferation of the intestinal epithelium. These mechanisms thereby regulate barrier function and minimize bacterial movement across the epithelium [21]. Additionally, zinc stimulates absorptive mechanisms that modulate ion transport, including exchange of Na^+^ and H^+^ ions, along with other transporters. Zinc also reduces net fluid loss and decreases stool volume, in diarrheal conditions, via inhibition of enterotoxin-stimulated secretory chloride flux [22]. Zinc also exhibits important immunomodulatory effects, as it aids in increasing innate immune cell function, works to support thymic hormone activity, promotes T-cell maturation, and is involved in cytokine response regulation. All these mechanisms collectively reduce enhanced pro-inflammatory signaling, while maintaining pathogen clearing activities. This reduces the risk and severity of any subsequent infections [23]. Zinc also depicts a bidirectional relationship with the gut microbiota and exhibits direct antimicrobial activity against enteric pathogens. It also impacts gut microbial composition in ways that favor diarrheal recovery [24]. These three mechanisms collectively explain the therapeutic significance of oral zinc intake in reducing duration of diarrhea, stool volume, and preventing diarrhea and respiratory disease recurrence in children living in low- and middle-income countries.

### 2.3. Zinc Absorption and Metabolism

Zinc absorption mainly takes place in the small intestine, especially in the duodenum and jejunum, through specialized carrier-mediated and saturable processes. The key transporter for bringing zinc from the intestinal lumen into the enterocytes is ZIP4, while ZnT-1 is responsible for exporting zinc from the enterocyte into the portal blood [25]. This coordinated action ensures that zinc is efficiently absorbed and made available to the body. About 37% of the zinc consumed is absorbed into plasma, with most of this absorption completed within four hours of ingestion. Once in the portal blood, the liver plays a major role by extracting around 67% of the absorbed zinc before it is released into the systemic circulation to reach other tissues [5]. The remaining zinc is then excreted in feces and varies between 0.8 and 2.7 mg/day. However, in zinc-deficient individuals, both fecal and urinary losses decrease rapidly. If the deficiency persists, the plasma zinc levels begin to decline, resulting in decreased amounts of exchangeable zinc in different tissues [26].

Beyond zinc absorption, metallothioneins regulate zinc homeostasis by binding excessive zinc and releasing it when needed. Zinc is also endogenously secreted into the intestine via sloughed off mucosal cells, bile, and pancreatic secretions. Zinc transportation is facilitated by transporters such a ZIP5, ZIP14, and ZnT5B. A majority of the zinc is bound to albumin and small amounts form linkages with α_2_-macroglobulin and transferrin [27]. The liver is the primary zinc reservoir and releases it into peripheral tissues when needed. The main zinc storage sites include the skeletal muscle (60%) and bones (30%), and the remaining zinc is stored in the skin and liver [28]. In states of zinc deficiency, the body may increase zinc absorption by up to 90% while tightly regulating its excretion through feces, urine, and skin [29].

The efficiency of zinc absorption can be influenced by dietary factors, such as the presence of phytates in plant foods, which can bind zinc and reduce its bioavailability. The body can adjust the expression of zinc transporters depending on zinc status, increasing absorption when zinc is low [5]. The IP6 forms a strong insoluble complex with zinc which cannot be absorbed by the enterocytes, whereas IP5 has a weaker inhibitory impact. Single meals with IP6 significantly reduce zinc absorption, whereas remaining phytates from meals can enhance zinc absorption [30]. However, zinc absorption is increased in the presence of dietary proteins, specifically animal proteins. The suggested mechanism states that proteins increase zinc solubility, which then enhances zinc absorption into the intestinal lumen. An example is milk citrate, which forms readily absorbed complexes with zinc, and because these complexes are found in higher concentrations in cow’s milk, zinc absorption is higher from cow’s milk as compared to human milk [29].

Furthermore, zinc bioavailability is further enhanced in the presence of polysaccharides because of their hydroxyl group which binds with metal ions such as zinc. This linkage forms a zinc–polysaccharide complex, which has higher solubility and stability. Additionally, these complexes prevent zinc from binding with dietary phytic acid, thereby reducing chelation. Due to the high solubility of the zinc–polysaccharide complexes, they are slowly released in the intestine, promoting their absorption and reducing zinc excretion via urine and feces. Research has suggested that enzyme-modified polysaccharides can further enhance zinc binding and subsequent bioavailability [31].

The effect of calcium on zinc absorption is nuanced and varies depending on dietary context. Earlier studies have suggested that calcium in the form of calcium carbonate or milk can reduce zinc intestinal absorption. However, when calcium is consumed with phytates, it forms a complex with phytic acid, thereby rendering it unavailable to bind with zinc. This enhances zinc absorption indirectly, despite calcium’s own zinc-binding properties. Studies combining milk with phytate rich foods found higher zinc bioavailability and absorption. The effect is also enhanced due to presence of milk citrates which further enhance zinc absorption; therefore, milk may have a higher impact on zinc bioavailability in comparison to calcium supplements [32].

Iron and zinc both compete for the same intestinal transportation pathway and hence influence each other’s absorption status. Non-heme, liquid, and ferrous iron all have an inhibitory impact on zinc absorption. However, iron-fortified foods and heme iron do not have any impact on zinc absorption or transport. Additionally, iron administered to pregnant females as a therapeutic dose also does not impact zinc absorption [33]. Dietary fibers also act as an anti-nutrient for zinc absorption, as they bind with it to form insoluble complexes which interfere with its absorption. When these fibers are fermented in the large intestine, they release the bound zinc. However, as the primary zinc absorption site is the small intestine, the effect of enteral fiber fermentation on zinc absorption is uncertain. Additionally, fibers may also increase intestinal viscosity, which hinders zinc and enzyme interaction. The type and dietary fiber source determine the overall inhibitory effect it will have on zinc absorption [34].

Dietary zinc absorption is further impacted by supplemental zinc, both of which compete via saturable absorption pathways for absorption. Therefore, increased dietary zinc intake can hinder the absorption of supplemental zinc [35]. Additionally, zinc intake and form both regulate absorption. When supplemental zinc is taken repeatedly, it may downregulate zinc transporters ZIP4 and ZnT1. This then disrupts natural zinc absorption and transportation mechanisms [9]. Moreover, as dietary zinc intake increases, the percentage of zinc absorption decreases. However, the net absorption still continues to increase slightly, which can be attributed to the saturation of zinc transporters, and reflects a non-linear pattern of absorption [36].

### 2.4. Zinc Recommendations (Doses and Side Effects)

For children under 5 years, the recommended dietary allowance is 3–5 mg of zinc daily to fulfill the requirements of nearly all healthy persons [11]. Current WHO guidelines recommend 20 mg daily for children aged 6 to 59 months with acute diarrhea for 10–14 days, and 10 mg per day for infants under six months, along with oral rehydration therapy [37]. However, recent studies suggest that lower doses may be equally effective with fewer side effects, particularly reduced vomiting. A multicenter trial found 5–10 mg/day of oral zinc to be equally effective in reducing diarrhea duration and stool output, as compared to 20 mg zinc per day [38]. A systematic review found that zinc supplementation reduced diarrhea duration by 13 h but also found a dose–response relationship between zinc intake and vomiting, as lower zinc doses were found to be associated with a 20% lower vomiting risk [39]. An Egyptian study compared the impact of different zinc doses (20 mg, 40 mg, and 60 mg daily), and found all doses to be effective, with faster recovery at higher doses, but fewer side effects at lower doses [40]; while the WHO still recommends 20 mg of oral zinc, emerging evidence has supported therapeutic doses of 5–10 mg per day for equivalent efficacy, highlighting important considerations for future policies (Table 3).

### 2.5. Causes and Prevalence of Zinc Deficiency in Different Regions

Zinc deficiency occurs due to a number of complex and interwoven factors, including lack of dietary zinc intake, certain physiological conditions, and environmental and socio-economic factors. Dietary inadequacy remains the topmost cause of zinc deficiency, particularly in children from low- and middle-income countries. This can be attributed to limited availability of zinc-rich animal foods, coupled with consumption of plant-based diets rich in phytates that further exacerbate deficiency risk [41]. Furthermore, socioeconomic factors aggravate financial constraints in accessing food, resulting in food insecurity and limited availability of zinc-rich foods. Population groups living below the poverty line often have to consume legumes and cereals on a daily basis, which are a poor zinc source and offer low zinc bioavailability [42]. Furthermore, in the case of malnutrition, zinc status becomes compromised as protein–energy malnutrition increases zinc requirements, while simultaneously increasing endogenous zinc losses [43]. Additional causes of zinc deficiency include increased demand for growth during the first year of life and early childhood, environmental factors such as poor zinc levels in soil, cultural or religious dietary restrictions limiting intake of zinc-rich foods, and certain rare genetic conditions that may cause zinc transporter protein defects [44].

Recent data on zinc deficiency emphasizes the high burden in children under five, especially those belonging to low- and middle-income countries. Globally, zinc deficiency fell from 40% in 1990 to 22% in 2020, among children under five. However, despite this decline in overall zinc deficiency burden, there remains within- and between-country variations, with resource-poor regions, like central Africa, exhibiting the highest prevalence [45]. At the population level, about 15–20% individuals worldwide are at risk of zinc deficiency, with a higher burden concentrated in low- and middle-income countries; however, certain population sub-groups in high income countries also demonstrate zinc deficiency risk. The African region reports the highest rates of zinc deficiency (20–24%), followed by South Asia and Southeast Asia (18–20%). Additionally, even higher rates are reported in specific settings in these countries, with zinc deficiency levels as high as 25–30% and above, stressing the need for targeted interventions to diminish zinc deficiency in these specific sub-groups [46]. Moreover, the morbidity and mortality rates attributed to zinc deficiency in all population groups have fallen in recent years; however, certain regions, including Central and East Africa, still report high deficiency statistics. Interventions targeting zinc deficiency should prioritize these areas to strive for complete eradication of zinc deficiency [47].

## 3. Pathophysiology of Diarrhea

### 3.1. Causes and Types of Diarrheas in Children

Diarrhea of childhood is a common clinical complaint and can be classified into several categories of pathophysiology: osmotic, secretory, inflammatory, and malabsorptive. Osmotic diarrhea occurs when unabsorbable solutes, in the case of undigested sugars with lactose intolerance, draw water into the lumen of the intestine. This type of diarrhea is typically cured by fasting. Secretory diarrhea, on the other hand, is caused by substances that stimulate the secretion of water and chloride into the intestine by the action of bacterial toxins like those of Vibrio cholerae or enterotoxigenic *Escherichia coli*. Secretory diarrhea, in contrast to osmotic diarrhea, persists even when the child has nothing to eat [11]. Inflammatory diarrhea results from damage to the intestinal mucosa, usually due to invasive organisms such as *Salmonella*, *Shigella*, or *Campylobacter* or chronic disease such as inflammatory bowel disease. It is often characterized by the presence of blood or mucus in the stool and systemic signs such as fever. Malabsorptive diarrhea happens when the intestine fails to properly absorb nutrients, as occurs in celiac disease or cystic fibrosis, and is typically associated with weight loss and loose, greasy stools [11].

There are several different reasons for diarrhea in children. Infectious causes remain the most common cause, and viruses, bacteria, and parasites all qualify as causative. Rotavirus-induced viral gastroenteritis is the primary cause of acute diarrhea and dehydration in children worldwide, particularly under the age of five years. Norovirus and adenovirus are also frequent perpetrators. Infections by *Escherichia coli*, *Salmonella*, *Shigella*, and *Campylobacter* are more likely to cause intense symptoms such as fever, abdominal cramps, and even bloody stools. Parasitic infections, such as Giardia lamblia and Cryptosporidium, are more likely to cause prolonged or chronic diarrhea, especially in low sanitation areas [48,49].

Non-infectious causes should also be considered, particularly recurrent or persistent diarrhea. Food intolerance such as lactose or fructose intolerance and food allergy such as cow’s milk protein allergy may cause chronic symptoms. Some other important causes include drug reactions (especially antibiotics that disturb the gut flora), and chronic gastrointestinal disease like celiac disease, cystic fibrosis, and inflammatory bowel disease. Even excess fruit juice intake or overfeeding in children can result in osmotic diarrhea [48].

Clinical presentations of diarrhea typically provide some clue regarding the cause. Acute infectious diarrhea is typically followed by vomiting, fever, dehydration, and abdominal cramps. Dehydration is a significant concern in young children due to their higher metabolic rate and lower fluid reservoirs. Severe or chronic bloody, mucus-containing diarrhea or systemic signs need to be immediately evaluated by a doctor to rule out severe illnesses like inflammatory bowel disease or surgical catastrophes [49].

### 3.2. Impact of Diarrhea on Child Development and Mortality

Diarrheal diseases are one of the leading causes of morbidity, mortality, and impaired cognitive and physical development in children under five. They contribute significantly to the death toll of children under five globally, with 9% of all deaths in this age group attributable to diarrheal diseases and associated complications. Additionally, approximately 1.6–1.7 diarrheal episodes are reported in children under five living in LMICs, annually [50]. Recurrent and chronic diarrheal episodes are associated with poor cognitive indicators in infancy and early childhood years. Diarrhea, if unresolved, can impair physical growth, resulting in stunting and long-term undernutrition that may have detrimental effects even through adulthood [51]. A majority of diarrheal diseases can be attributed to rotavirus infection, which results from a category of enteric pathogens, and mainly affects infants and children under five. However, since efforts have increased in reference to the administration of rotavirus vaccines, diarrheal episodes and diarrhea-related deaths have significantly dropped. However, diarrheal mortality burden still remains high in resource-poor regions with low rotavirus vaccine coverage. In developed countries, like the USA, rotavirus-attributed diarrheal hospitalizations have been reduced substantially; however, diarrhea attributed to norovirus and residual cases of rotavirus diarrhea still contribute to emergency visits among infants and young children [52]. These statistics highlight the need to widen diarrheal preventive strategies, including hand washing, oral rehydration therapy, zinc supplementation, and proper nutrition, to reduce the risk of associated mortality.

### 3.3. Role of Zinc in Gastrointestinal Health and Diarrhea

Zinc plays a critical role in maintaining innate and adaptive immune systems at the cellular level, and insufficiency leads to immune cells developing and reacting abnormally, disrupting intracellular communication necessary for coordinated immunity defense [11]. Zinc is vital for maintaining intestinal mucosal integrity, regulating junction proteins, and contributing to epithelium repair. These mechanisms help prevent pathogen entry into the gut, and limit fluid losses during enteral infections [53]. Beyond zinc’s therapeutic role in reducing the duration and severity of diarrheal illness, its deficiency is associated with gut dysbiosis, alteration of host and microbiota interactions, and enhancement of gut permeability. All these mechanisms are subsequently tied to acute and chronic gut disorders. Adequate zinc intake has been reported to have prevented the majority of diarrheal deaths annually in LMICs. Zinc’s role in maintaining the gut barrier highlights its significance beyond its role as an immune-modulatory nutrient [54].

The positive correlation between zinc supplementation and better diarrheal outcomes in children is supported by a sizable body of solid research [12]. Ali et al. (2024) confirmed the effectiveness of zinc supplementation in treating acute and chronic diarrheal episodes by conducting a systematic review and meta-analysis of 38 randomized controlled trials [55]. They observed that lower dosages were associated with a lower frequency of side effects, including emesis. Meanwhile, in a cohort of 140 children, El-Ghaffar et al. (2022) reported statistically significant decreases in the incidence and recurrence of diarrheal illness after four months of daily zinc supplementation [56]. Additionally, Dhingra et al. (2020) demonstrated that the therapeutic benefits of lower zinc dosages (5, 10, and 20 mg) were not inferior to higher dosages and demonstrated superior tolerability, as evidenced by a lower prevalence of emesis in a large-scale randomized controlled trial with 4500 pediatric participants [57].

Additionally, recent evidence suggests that zinc supplementation is the most effective in reducing the duration and severity of diarrheal diseases in population groups with the highest baseline zinc deficiency [58]. Furthermore, zinc supplementation has greatly reduced diarrhea-associated morbidity and mortality in several community-based trials, underscoring its importance in LMICs’ infant and young child programs [59]. A meta-analysis on zinc supplementation and its role in preventing diarrhea reported a 16% reduction in all-cause mortality risk in children. Additionally, it was reported that consumption of 10 mg per day or more of zinc for less than 11 months decreased all-cause mortality by 44%. Diarrhea-specific mortality reduction was reported to be 15% after less than a year of zinc supplementation [60]. Intervention studies have reported a combination of both zinc and oral rehydration solution (ORS) within routine diarrheal management to be cost-effective and efficient in reducing child mortality burden [61].

Supplementing with zinc has been shown to successfully reduce the intensity and length of acute diarrheal episodes in children [12]. A systematic review of 38 randomized controlled trials found that zinc treatment was associated with higher clinical resolution rates and an average decrease in diarrheal episode duration of about 13.27 h compared to placebo treatment [12]. Furthermore, convergent data indicates that zinc helps to resolve clinical symptoms more quickly; the control group’s mean recovery duration was 4.74 days, whereas the zinc-supplemented group’s was 3.34 days [49].

## 4. Zinc Deficiency and Risk of Diarrhea

Zinc deficiency significantly compromises gut barrier function through a range of interconnected mechanisms that impact both the physical and immune defenses of the gastrointestinal tract [11]. One of the primary ways in which zinc deficiencies impair gut integrity is by suppressing the secretion of secretory immunoglobulin A (sIgA) in the intestinal tract, which occurs due to the negative effects on gut-associated lymphoid tissue (GALT) function [5,62]. Secretory IgA is a crucial component of the intestinal immune barrier, acting as the first line of defense against pathogens by neutralizing toxins and preventing microbial adhesion to the mucosal surface. When IgA levels are reduced, the intestinal barrier becomes more vulnerable to invasion by harmful bacteria and viruses, increasing the risk of gastrointestinal infections [11].

Furthermore, zinc plays a vital role in maintaining the structural integrity of the intestinal epithelium. Zinc deficiency disrupts the formation and maintenance of tight junctions between epithelial cells, leading to increased intestinal permeability or “leaky gut” [5,63]. This breakdown in barrier function allows pathogens and toxins to translocate across the gut lining more easily, which can trigger local and systemic inflammatory responses. Animal and in vitro studies have shown that zinc supplementation can restore tight junction integrity and improve barrier function, highlighting its essential role in gut health [63].

In addition to weakening the physical barrier, low zinc levels increase susceptibility to both bacterial and viral diarrhea by impairing multiple aspects of immune function [48]. Zinc deficiency has been shown to induce inflammatory responses via bacterial translocation to the liver through the portal vein, a process that further demonstrates how compromised gut barrier function can lead to systemic complications and inflammation [5,62]. This can result in a cycle of gut–liver axis dysfunction, perpetuating immune dysregulation and increasing the risk of severe infections.

Children suffering from zinc deficiency are at a much higher risk of developing infectious diseases, particularly diarrheal illnesses. Epidemiological data indicate that zinc deficiency is responsible for approximately 453,207 deaths annually (4.4% of childhood deaths) from diarrhea, malaria, and pneumonia in Africa, Asia, and Latin America [4]. Of these, a significant proportion is directly attributable to diarrhea, with zinc deficiency accounting for 14.4% of diarrhea deaths, 10.4% of malaria deaths, and 6.7% of pneumonia deaths among children between 6 months and 5 years of age [4]. These figures underscore the critical importance of adequate zinc status for maintaining gut barrier integrity, supporting immune defense, and ultimately reducing preventable childhood mortality.

## 5. Zinc Dosage and Administration During Diarrhea

### 5.1. WHO Guidelines: Age-Specific Dosages

The World Health Organization and United Nations Children’s Fund suggest dosage by age for zinc supplementation during the duration of an acute attack of diarrhea. Children two months and above need 20 mg per day for 10–14 days and 10 mg per day for children below six months [64]. These suggestions are not only designed to reduce the duration and intensity of this present bout of diarrhea but also to forestall subsequent bouts for the following two to three months [11]. Zinc supplementation has also been shown to reduce the duration of diarrhea by approximately 25% and decrease stool volume by approximately 30% [65]. The WHO also emphasizes zinc is particularly beneficial in settings with high rates of zinc deficiency and supports its use as an integral part of standard management in every child with acute diarrhea [65,66].

### 5.2. Duration and Formulation Types

Acute diarrheal disease is typically managed by 10–14 days of treatment, where zinc and ORS are the cornerstones of treatment to reduce severity, duration, and risk of dehydration [37,65]. Current data show that doses of 5 mg or 10 mg of zinc are as effective as the accepted dose of 20 mg with less in the way of side effects such as vomiting and are thus the treatment of choice for the majority of children [14]. Zinc preparation selection, i.e., syrups, dispersible tablets, or plain tablets, can affect acceptability and palatability, especially among young children [11]. Acute and chronic diarrhea continue to require ORS to prevent dehydration and refeeding early with appropriate food, including continued breastfeeding, is recommended to facilitate recovery and prevent malnutrition [65].

Chronic diarrhea, which persists for more than four weeks, requires a different approach involving searching for and treating the underlying cause [67,68]. Management may consist of dietary modification, such as the elimination of causal foods in food intolerance (e.g., lactose, gluten) or allergy and nutritional replenishment to break the vicious circle of diarrhea and malnutrition [67,68]. Chronic infections may be treated with antibiotics or antiparasitic medication, and inflammatory disorders like inflammatory bowel disease may be managed with anti-inflammatory agents or immunosuppressives [67]. Functional gastrointestinal diseases, like toddler’s diarrhea or irritable bowel syndrome, are usually managed with water intake, dietary changes, and reassurance, as most children will recover from these diseases [67]. In all cases, hydration with ORS and waiting patiently for dehydration are critical aspects of treatment [69].

### 5.3. Strategies to Improve Adherence

Improved adherence to zinc supplementation is crucial for the successful management of the disease. Side effect control, particularly vomiting, which is exacerbated at higher doses, can enhance compliance [12]. Low-dose regimens have also been found to reduce vomiting and, in turn, enhance compliance [14]. The palatability of the drug, for instance, through syrups or dispersible tablets, may also enhance better swallowing in children in line with the dosage as prescribed [11]. Furthermore, ORS use, together with zinc and other supportive treatment, is proven to improve outcomes and acceptability [64,65]. Educating caregivers on the importance of completing the full course, recognition of dehydration signs, and good hygiene practices are basic steps to further improve adherence and reduce recurrence risk [65].

## 6. Long-Term Benefits of Zinc Supplementation for Diarrhea

### 6.1. Reduced Incidence of Future Diarrheal Episodes

Zinc supplementation also has protective effects within treatment periods of short duration [70]. Zinc therapy has been shown to reduce the duration and severity of diarrheal episodes for three months following therapy [11]. Martinez-Estevez et al. (2016) demonstrated that low-dose zinc supplementation of 5 mg/day for 12 months was effective in preventing diarrhea and upper respiratory infection [70].

### 6.2. Improved Linear Growth and Nutritional Recovery Post-Diarrhea

Several growth benefits of zinc supplementation were described in some studies [71]. Islam et al. (2022) [26] found superior growth with high-zinc, low-iron micronutrient powder in their six-arm RCT of 2886 children. Impacts on growth outcomes have been inconsistent, with Barffour et al. (2018) finding better zinc status but no growth effect in their trial of 3407 children [72].

### 6.3. Broader Impact on Child Development and School Readiness

Even if direct evidence from the studies analyzed for school preparedness is limited, prevention of diarrheal attacks and associated malnutrition will likely lead to enhanced cognitive development and school enrollment [4]. Minimization of the disease burden from diarrhea and respiratory infections permits children to have more opportunities for typical development and learning [70].

## 7. Intervention Strategies

### 7.1. Zinc Supplementation

Therapeutic zinc supplementation during acute diarrheal episodes is strongly supported by a substantial body of evidence, highlighting its critical role in pediatric diarrhea management [12]. Multiple randomized controlled trials and systematic reviews have consistently demonstrated that administering zinc supplements to children with diarrhea significantly reduces both the duration and severity of diarrheal episodes [64]. The recommended WHO zinc supplemental dosage is 10–20 mg elemental zinc per day for 2 weeks (14 days). Evidence has found this dosage and duration sufficient to produce clinical benefits, aid in diarrhea recovery, and reduce diarrhea duration by 13.3 h, as compared to a placebo. Additionally, moderate zinc doses (5–10 mg per day) have demonstrated similar efficacy to the 10–20 mg per day dosage but with lower vomiting risk [3]. Zinc supplementation has proven effective not only for acute diarrhea but also for persistent cases, with studies showing improved recovery rates and faster symptom resolution in children receiving zinc compared to those who do not [64]. Moreover, lower doses of zinc have been found to be as effective as higher doses, while causing fewer side effects such as vomiting, making adherence easier for children and caregivers [14]. For preventive purposes, a daily dose of 10–15 mg elemental zinc is recommended, as it is associated with modest decreases in diarrhea incidence; however, this dosage was also linked with vomiting episodes [22]. The recommended dosage for pre-term and very low birth weight infants is 3–5 mg per day, which is linked with body weight and length improvements, as well as reduced diarrheal episodes [60]. There is growing evidence that zinc oxide nanoparticles may offer greater benefits as compared to standard zinc supplementation. This is attributed to the zinc nanoparticles’ ability to improve intestinal barrier function and restore tight-junction integrity during enteral inflammatory conditions. These nanoparticles also strengthen the gut’s antioxidant defense, thereby enhancing zinc delivery and efficacy [40].

The World Health Organization and UNICEF endorse zinc supplementation dosing at 10–20 mg per day, as a standard of care for childhood diarrhea, emphasizing its importance in reducing morbidity and preventing subsequent episodes. However, lower doses at 5–10 mg per day may also exhibit similar efficacy, without adverse effects. While zinc supplementation is generally safe, side effects may occur with doses higher than 5–10 mg per day. The most noticeable side effect is vomiting, with children under five having a 46% higher risk of vomiting, which subsided as zinc doses dropped [72]. Additionally, an RCT on 4500 children receiving zinc supplementation revealed that being of a younger age, undernourished, and dehydrated further increased vomiting risk in children under five. In addition to gastrointestinal distress, zinc supplementation at doses higher than 10–20 mg/day for infants, and 20–30 mg/day for toddlers, was linked with regurgitation and dysguesia leading to lower appetite and compromised dietary intake [8].

However, drugs and supplements used for treatment of infectious diseases in children including tablets, gums, capsules, and syrups can differ in formulations, active drug consistency, and compliance. Different zinc salts at low, pediatric, palatable, or easy-to-administer doses are not always available at all geographic locations, which can be challenging for the pharmacological industry and for a clinical setting [53].

### 7.2. Zinc Fortification

Large-scale food fortification is recognized as a powerful, population-level strategy to prevent zinc deficiency and its associated health consequences [5]. Unlike individual supplementation, fortification involves adding zinc to staple foods such as cereal grains, ready-to-eat meals, and micronutrient powders, thereby increasing zinc intake across entire communities. This intervention is particularly valuable in regions where dietary zinc intake is consistently low due to limited access to animal-source foods. Evidence suggests that implementing high-quality, mandatory fortification programs that include zinc could reduce the estimated prevalence of inadequate zinc intake by up to 50% globally, making a significant impact on public health [5]. For example, fortification strategies in Bangladesh and other countries have included the addition of zinc to wheat flour, rice, and other staple foods, as well as the use of micronutrient powders for home fortification [71]. While fortification alone may not eliminate zinc deficiency, it can substantially improve dietary zinc intake and related health outcomes, especially when combined with other nutrition interventions.

Recent evidence has framed zinc fortification of cereal and rice as a breakthrough in reducing zinc deficiency by 30 to 50% in LMICs, particularly in South Asia and Sub-Saharan Africa [70]. A randomized controlled trial from Pakistan found zinc-fortified wheat flour to increase zinc intake per day (1.5 mg/day), which was a 21% increase as compared to the control group. These findings have important implications in improving zinc plasma and serum concentrations, especially among under-privileged women and children [20]. Additionally, home fortification of zinc-containing micronutrient powders has also been evidenced to decrease diarrheal episodes and improve growth outcomes in children. The WHO recommends home micronutrient powders with 4.1–5 mg of zinc, 12.5 mg of iron, and 13 other important micronutrients [6]. Importantly, the food vehicle for zinc fortification plays a major role in fortification effectiveness, as it influences coverage and zinc bioavailability. Various pathological conditions, such as infections, and environmental factors, like dietary diversification, also impact the effectiveness of zinc fortification interventions [65]. As per the Global Fortification Data Exchange, currently 20 countries around the world have mandated zinc fortification of at least one staple food; while this intervention has substantially improved zinc deficiency statistics globally, coverage gaps still remain in LMICs in areas like Africa and Southeast Asia [19].

### 7.3. Combined Interventions

Combined interventions that integrate zinc supplementation with other micronutrients or treatments have shown promise for enhanced effectiveness in managing and preventing diarrhea in children [37]. For instance, studies have found that the combination of vitamin A and zinc supplementation yields optimal improvements in outcomes for children with persistent diarrhea, with the group receiving both nutrients experiencing the greatest reduction in symptom duration and improved nutritional status. Additionally, the co-administration of zinc with new low-osmolarity ORS has been shown to further reduce the duration and severity of diarrheal episodes, offering a synergistic benefit [64]. These integrated approaches not only address acute illness but also help prevent future episodes and improve overall child health. The evidence suggests that comprehensive strategies, which include zinc supplementation, vitamin A, ORS, and other established interventions like breastfeeding and hygiene, are most effective in reducing the burden of childhood diarrheal disease.

### 7.4. Public and Community Zinc Awareness for Diarrhea in Children

Public awareness campaigns are essential for the successful implementation and uptake of zinc interventions in the community [5]. Education of healthcare providers, caregivers, and the broader public about the benefits of zinc supplementation during diarrheal episodes is crucial for improving adherence to treatment protocols and ensuring that children receive the recommended care [11]. Effective campaigns often use multiple communication channels, including television, radio, outdoor advertising, and community outreach, to increase knowledge and acceptance of zinc treatment. Studies have shown that such campaigns can rapidly raise awareness and usage rates, although continued efforts are needed to overcome barriers related to access, cultural beliefs, and health system limitations. Ultimately, increasing public awareness and education about zinc’s role in diarrhea management is a key component of comprehensive strategies to reduce childhood morbidity and mortality from diarrheal diseases.

## 8. Risk Factors and Barriers to Zinc Intervention

### 8.1. Cultural and Behavioral Practices

Health belief models and sociocultural factors can have a significant impact on how zinc supplementation strategies are used and integrated [5]. Programmatic implementation may be hampered by a preponderance of local treatment methods and concerns about traditional biomedical technologies [11]. Therefore, it is crucial to use culturally appropriate health communication techniques and community involvement to overcome these obstacles and encourage the successful implementation of zinc supplementation programs [5].

### 8.2. Food Accessibility and Insecurity

Nutritional insecurity is a significant barrier to obtaining zinc-rich foods and can thus undermine the overall effectiveness of supplementation programs [5]. People who purchase fewer animal-based foods are more likely to be zinc-deficient because they consume primarily plant-based diets with naturally lower zinc levels [72].

### 8.3. Supplementation Acceptance and Compliance Issues

The administration of zinc supplementation protocols may be complicated by adverse effects, predominantly manifesting as emesis [12]. Clinical research findings show that children who take zinc have a noticeably higher risk of emesis than children who receive a placebo [14]. In order to improve patient adherence, attenuated-dose regimens that maintain therapeutic effectiveness while reducing gastrointestinal side effects may be developed [12].

### 8.4. Supply Chain and Pragmatic Limitations

Weak supply chains and lack of consistent availability of zinc supplements, particularly in LMICs, often contribute to limited coverage and sustainability of supplementation interventions. Evidence has reported a lack of zinc supplementation at healthcare facilities and inadequate distribution channels, contributing to poor caregiver access, despite zinc supplementation being a part of national guidelines. This results in poor treatment and low coverage of zinc therapy, resulting in unchanging diarrheal burden [59]. Therefore, it is vital to strengthen logistics and ensure uninterrupted access to zinc supplementation in order to overcome this availability barrier.

### 8.5. Knowledge Gaps Among Healthcare Providers and Caregivers

Frontline healthcare workers are usually the ones dealing with infants and children presenting with severe diarrheal illness. Their lack of awareness and training regarding administering zinc supplementation as a management strategy for diarrhea prevention and management contributes to low utilization of zinc supplementation. Studies from Africa and South Asia have reported failure to prescribe zinc supplementation consistently with ORS as a treatment regimen for diarrhea. This has been attributed to lack of knowledge about its evidenced benefits [47]. Similarly, caregivers with low levels of education are less likely to administer an ORS plus zinc therapy for managing their child’s diarrheal episodes, and in contrast, the more knowledgeable they are, the more they actively practice ORS with zinc supplementation. Therefore, health education and awareness creation in mothers and caregivers are crucial for enhancing zinc supplementation efforts [17].

## 9. Conclusions

Zinc deficiency is a significant public health problem and remains so, especially among children in low- and middle-income countries, and is a significant cause of diarrheal morbidity and mortality. The evidence is established that zinc supplementation reduces the duration and severity of acute and persistent diarrhea among children, with the greatest impact observed in malnourished children. Preventive strategies, including mass food fortification and targeted supplementation, are recognized as prominent public health responses to address widespread zinc deficiency and its associated health consequences. There is now sufficient evidence to call for zinc supplementation as a routine component of the treatment of children with diarrhea, as taken up by the World Health Organization and UNICEF. Proposed regimens of 10–20 mg/day for 10–14 days have been shown to hasten recovery, reduce stool volume, and reduce the likelihood of subsequent diarrheal illness. Interestingly, lower-dose regimens are proving to be effective with less adverse effects, which should improve compliance and overall success. These advances notwithstanding, some challenges remain. Uncertainty about the best dosing regimens, delivery systems, and sustained delivery in resource-poor, heterogeneously structured settings remains. Food insecurity, culture, and side effects such as vomiting pose barriers to effective zinc interventions. They call for culturally adapted health education, community participation, and further research aimed at further focusing intervention strategies. In general, zinc supplementation and fortification are cost-saving, evidence-based strategies to reduce the morbidity of childhood diarrhea and promote improved child health outcomes globally. This is a comprehensive review focusing on the importance of zinc for diarrhea treatment in children. However, we could not find enough studies about different zinc salts at low, pediatric, palatable, and easy-to-administer doses available in all geographic locations, which can be considered as a study limitation. More work must be performed to optimize dosing regimens, improve delivery systems, and incorporate zinc interventions into large child health and nutrition programs so that their full potential can be tapped. Future research must explore modified zinc supplementations such as nanoparticles for more efficient delivery and absorption and enhanced therapeutic effects. Understanding interactions between zinc and other nutrients as well as other forms of zinc may also help optimize the mode of zinc supplementations. Interventions aimed at promoting zinc intake must be culturally tailored to local dietary patterns and health system logistics to enhance the effectiveness of zinc interventions.

## Figures and Tables

**Table 1:** Biological role of zinc in children’s growth and immunity.

Biological Function of Zinc	Mechanism of Action	Outcomes in Children
Anti-inflammatory	Inhibits Nuclear Factor-kappa B (NF-κB) activation Limits histamine release from basophils, leukocytes, and mast cells	Decreased pro-inflammatory cytokines Reduced tissue damage from excessive immune response
Antioxidant defense	Acts as a cofactor for superoxide dismutase Regulates oxidative balance Upregulates protective factors like A20-TNFAIP3 and IKBα	Protection against oxidative stress Reduced reactive oxygen species (ROS) Decreased release of inflammatory cytokines
Growth and development	Supports protein, lipid, and nucleic acid metabolism Maintains cell integrity and proliferation	Supported normal growth and neurocognitive development Prevents stunting
Innate immunity	Regulates neutrophil and natural killer (NK) cell activity Controls intracellular immune signaling pathways	Improved first-line defense against infections
Adaptive immunity	Essential for lymphocyte maturation, antibody production Regulates cytokine signaling	Stronger acquired immunity Improved pathogen response
Thymulin function	Supports thymulin hormone activity needed for T-cell maturation	Adequate T-cell differentiation Correction of immune defects in zinc-deficient children

IKBα—Inhibitor of Nuclear Factor-kappa B alpha. A20-TNFAIP3—Tumor Necrosis Factor Alpha-Induced Protein 3.

**Table 2:** Therapeutic mechanisms of zinc in childhood diarrheal diseases.

Pathway	Mechanism of Action	Therapeutic Effect in Diarrhea
mucosal/epithelial repair	Acts as a cofactor for enzymes and zinc-finger proteins Promotes cell proliferation, DNA synthesis, and apoptosis regulation. Stabilizes enterocyte membranes Reduces intestinal permeability Increases epithelial regeneration.	Restored intestinal barrier integrity Reduced bacterial translocation
Ion Transport and Fluid Balance	Stimulates Na^+^/H^+^ exchange and other absorptive transporters Inhibits enterotoxin-induced chloride secretion	Reduced fluid loss Decreases stool output Reduces diarrhea severity
Immune Modulation	Supports thymic hormone (thymulin) Promotes T-cell maturation Regulates cytokine Increases innate immune cell function	Decreased pro-inflammatory signaling Maintained pathogen clearance
Gut Microbiota Interaction	Direct antimicrobial activity against enteric pathogens Modulates microbiota composition to favor recovery	Reduced pathogen load Improved microbial balance during recovery

**Table 3:** Recommended doses of zinc per age group and condition.

Age Group	Condition	Recommended Dose and Duration
Infants < 6 months	RDA (healthy)	3 mg/day
	Acute diarrhea	10 mg/day for 10–14 days (with ORS)
Children 6–59 months	RDA (healthy)	5 mg/day
	Acute diarrhea (WHO recommendation)	20 mg/day for 10–14 days (with ORS)
	Acute diarrhea (lower limit)	5–10 mg/day

## Data Availability

Not applicable.
